# A catalog of curated breast cancer genes

**DOI:** 10.1007/s10549-021-06441-y

**Published:** 2021-11-10

**Authors:** Muthiah Bose, Jan Benada, Jayashree Vijay Thatte, Savvas Kinalis, Bent Ejlertsen, Finn Cilius Nielsen, Claus Storgaard Sørensen, Maria Rossing

**Affiliations:** 1 Biotech Research and Innovation Centre (BRIC), Faculty of Medical and Health Sciences, University of Copenhagen, Copenhagen, Denmark; 2 Centre for Genomic Medicine, Rigshospitalet, Copenhagen University Hospital, Blegdamsvej 9, 2100 Copenhagen, Denmark; 3 Department of Clinical Oncology, Rigshospitalet, Copenhagen University Hospital, Copenhagen, Denmark; 4 Department of Clinical Medicine, University of Copenhagen, Copenhagen, Denmark

**Keywords:** Breast cancer, Genetic predisposition, Rare-monogenic variants, Common-polygenic variants, Database, DNA repair pathways

## Abstract

**Purpose:**

Decades of research have identified multiple genetic variants associated with breast cancer etiology. However, there is no database that archives breast cancer genes and variants responsible for predisposition. We set out to build a dynamic repository of curated breast cancer genes.

**Methods:**

A comprehensive literature search was performed in PubMed and Google Scholar, followed by data extraction and harmonization for downstream analysis.

**Results:**

Using a subset of 345 studies, we cataloged 652 breast cancer-associated loci across the genome. A majority of these were present in the non-coding region (i.e., intergenic (101) and intronic (345)), whereas only 158 were located within an exon. Using the odds ratio, we identified 429 loci to increase the disease risk and 198 to confer protection against breast cancer, whereas 25 were identified to both increase disease risk and confer protection against breast cancer. Chromosomal ideogram analysis indicated that chromosomes 17 and 19 have the highest density of breast cancer loci. We manually annotated and collated breast cancer genes in which a previous association between rare-monogenic variant and breast cancer has been documented. Finally, network and functional enrichment analysis revealed that steroid metabolism and DNA repair pathways were predominant among breast cancer genes and variants.

**Conclusions:**

We have built an online interactive catalog of curated breast cancer genes (https://cbcg.dk). This will expedite clinical diagnostics and support the ongoing efforts in managing breast cancer etiology. Moreover, the database will serve as an essential repository when designing new breast cancer multigene panels.

**Supplementary Information:**

The online version contains supplementary material available at 10.1007/s10549-021-06441-y.

## Background

Breast cancer is the most common cancer diagnosed in women and most importantly, it is the leading cause of cancer-related deaths among women worldwide [1, 2]. Breast cancer is a multifactorial disease resulting from genetic, hormonal, and environmental factors. In concordance with cancer disease in general, inherited mutations play a causal role in up to ten percent of all breast cancers [3, 4]. For decades, genetic screens have played a vital role in the identification of genes and variants responsible for breast cancer predisposition. Various sequencing methods such as Sanger sequencing, gene panel testing, whole-exome sequencing (WES), and ultimately whole-genome sequencing (WGS) have been employed to identify genetic variation responsible for breast cancer predisposition [5, 6]*.*

Genetic variation can predispose to breast cancer through both rare-monogenic variant causing a large increase in disease risk and common-polygenic variant (alias SNPs) that possess small individual effects on disease, however, cumulatively cause a large increase in disease risk [7]. Rare germline variants in the high-risk genes *BRCA1* and *BRCA2* together with the moderate-risk genes such as *PALB2*, *ATM*, *CHEK2,* and *BRIP1* account for about 30% of breast cancer predisposition [8, 9]. Similarly, cancer syndrome genes (*CDH1, PTEN* and *STK11* etc.) together with SNPs explain around 20% of breast cancer predisposition [9]. Most SNPs are identified through genome-wide association studies (GWAS) and recent studies have suggested that polygenic risk score (PRS) accounts for around 18% of the familial breast cancer risk [10]. The remaining heritability (around 50%) for breast cancer is most likely caused by yet unidentified moderate-risk genes or a specific cluster of common-polygenic variants [11]. Identification of these unknown factors responsible for breast cancer etiology is of utmost importance and could expedite personalized breast cancer medicine, including therapeutic and preventive strategies [12].

Breast cancer genes and SNPs responsible for disease etiology play a significant role during clinical management. The clinical utility of rare-monogenic variant containing genes and SNPs differs due to varied disease penetrance. Specifically, rare-monogenic variant containing breast cancer genes is used to design (or update) a focused panel of breast cancer genes for genetic screening. Similarly, a list of such genes could ensure that the clinical investigators has an updated breast cancer gene list, when screening patients for disease etiology using WES or WGS. For clinical purposes, the use of breast cancer genes and SNPs can be augmented significantly by rapidly integrating newly identified breast cancer genes and variants. However, with a constant flow of new studies, it is challenging to seamlessly translate these findings into the clinical setting. We believe the presence of a freely accessible database comprising breast cancer-associated genes (and variants) will aid a rapid translation into clinical diagnostics. This led us to initiate a meta-analysis of breast cancer susceptibility genes, by applying comprehensive and stringent criteria, with the aim of generating an online interactive catalog of curated breast cancer genes (https://cbcg.dk).

## Materials and methods

### Literature search and study selection

**Fig. 1:**
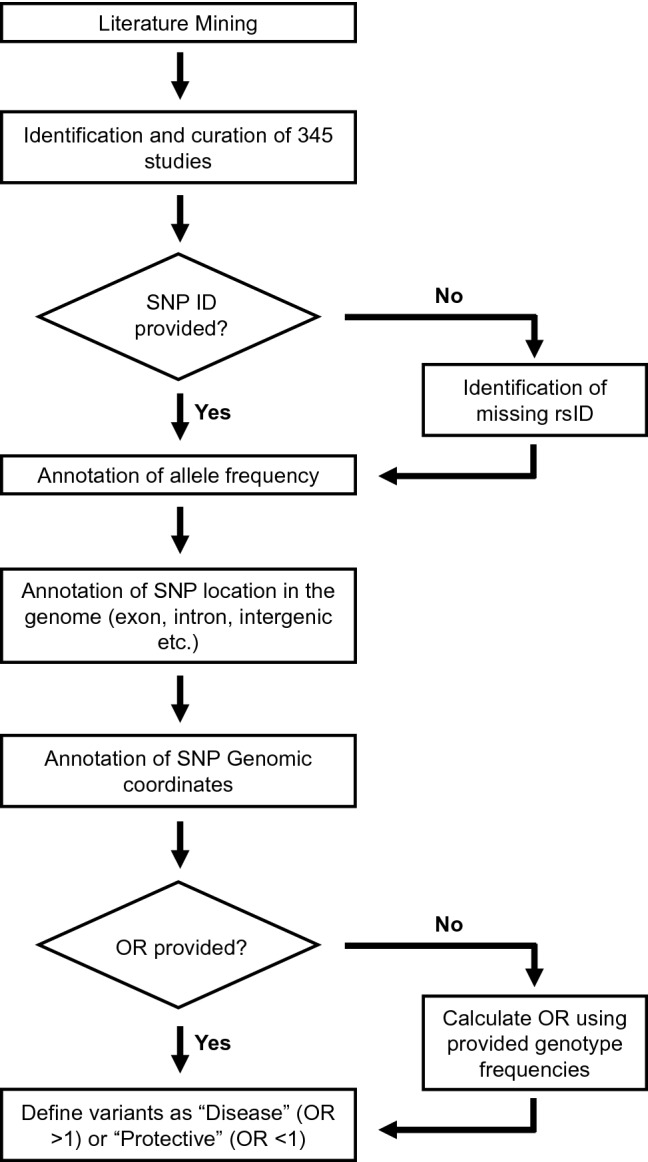
Flow chart outlining multiple steps involved in the database design such as literature search, data extraction, data annotation, and data harmonization

A comprehensive literature search for eligible studies was performed in PubMed and Google Scholar (Fig. 1). The following terms were used either alone or in combination: “Breast cancer”, “risk”, “loci”, “single nucleotide polymorphism”, “SNP”, “polymorphism”, “susceptibility gene”, “genetic variants”, “association”, “polymorphisms”, “genetic mutation”, “germline”, and “variant”. The inclusion criteria for the studies were as follows: (1) studies must be reported in English; (2) studies must be published in peer-reviewed journals; (3) studies must be available as full-text articles; (4) studies must be either case–control, kin-cohort, or prospective in design; (5) case–control studies must report genotype frequencies (or OR with 95% confidence interval (CI) values); and (6) for non-case–control study in design, other relevant metrics such as standardized incidence ratios (SIR), relative risk and cumulative risk etc. were taken into account (Fig. 1). The exclusion criteria were as follows: (1) publications that were reviews, meta-analysis, case reports, and meeting abstracts; (2) studies that did not provide genotype distributions among cohorts; and 3) studies performed on tumor tissue for breast cancer association (Fig. 1).

### Data extraction

Three independent investigators extracted all data and any discrepancies were resolved by discussion. The following information were collected from the enrolled studies: (1) SNP identifiers (rsID) (if reported) or the sequence variation of the reported mutation, (2) OR (if reported) or the genotypic frequency of both cases and controls, and (3) in relevant studies: SIR, relative risk, and cumulative risk were also collected (Fig. 1). Of note, information on population background and breast cancer subtypes was initially extracted from the enrolled studies. However, due to ambiguous use of descent, ethnicity, and nationality, as well as lack of consistent subtype annotation, these records were not included in the database.

### Data harmonization

Historically, different nomenclatures have been used to report the findings among the included studies (Fig. 1). Specifically, few studies have reported the breast cancer-associated variant using its rsID, whereas other studies reported only the consequent “sequence variation”. Similarly, few studies reported the OR of the identified breast cancer-associated variant (with 95% CI values), whereas other studies only reported the genotype frequencies between their study subjects. Thus, in order to standardize the data for this database, we performed data harmonization as shown in Fig. 1. The breast cancer-associated mutation that was reported only by its “sequence variation” was manually converted into its corresponding “rsID” using GnomAD database. Similarly, in those studies, which only reported the genotype frequencies between their study subjects, we manually calculated the corresponding OR (Fig. 1). The odds ratio, its standard error, and 95% confidence interval are calculated according to Altman, 1991 [13]. Specifically, OR is calculated using the formulae: OR = (a/b)/(c/d); where a = number of patients in disease cohort carrying the variant; b = number of patients in disease cohort not carrying the variant; c = number of patients in control cohort carrying the variant; and d = number of patients in control cohort not carrying the variant.

### Database design

This database was designed and created using the rsID and OR that were extracted as mentioned above (Fig. 1). Using the rsID, we manually annotated its allele frequency (AF), SNP location, and genomic loci. AF (GnomAD [14]) was used to differentiate the rare-monogenic variants (AF < 0.01) between the common-polygenic variants (AF > 0.01). SNP location illustrates whether a variant is located within a gene (intron, exon or UTR region etc.) or in an intergenic region. Genomic loci categorize both chromosomal regions with high clustering of breast cancer-associated mutations, as well as chromosomal segments that are devoid of breast cancer-associated mutation. The OR (also SIR, relative risk and cumulative risk) was used to differentiate between a potentially disease-causing genetic variant (OR > 1; hereafter referred as disease variant) and a genetic variant that may confer protection against breast cancer (OR < 1; hereafter referred as a protective variant).

### Chromosomal ideogram visualization

Chromosomal ideogram was constructed with PhenoGram software tool [15] (visualization.ritchielab.org) using proximity algorithm for phenotype spacing, with each circle representing one gene or variant. For the clarity of visualization, genomic coordinates were rounded to the nearest multiple of 1 Mb, and thus, genes or variants within this proximity were binned to a single line of adjacent circles. Final graphical adjustments were performed in Adobe Photoshop 2019 and Adobe Illustrator 2019.

### Network analysis

The protein–protein interaction network was constructed using STRING version 11.0 database [16] (https://string-db.org/). "Experiments" dataset was used as an active interaction source with a minimum required interaction score of 0.4 (medium confidence). Subsequently, the network visualization was graphically adjusted in Cytoscape 3.8.2 [17] (https://cytoscape.org/) in order to highlight proteins encoded by DNA repair genes.

### Functional enrichment analysis

g:Profiler [18], g:GOSt tool, was used to perform functional enrichment analysis in the breast cancer-associated genes resulting in a list (Supplementary Table 1) of 2068 significantly enriched terms (Benjamini–Hochberg FDR < 0.05). The gene list was treated as an unordered query and only annotated genes were considered for statistical tests under the statistical domain scope function. For term sizes, between 4 and 500 genes were considered. Electronic GO annotations were removed, while GO molecular function (MF), GO cellular component (CC), GO biological process (BP), KEGG, Reactome, and WikiPathways data sources were analyzed. The Ensembl ID with the most GO annotations was chosen for all 5 ambiguous genes (*AHRR*, *BABAM1*, *FOXP1*, *LRTOMT,* and *SULT1A1*).

## Results

### Literature search, data extraction, and annotation

The presence of genetic risk factors and positive family history of breast cancer is the single most important risk factor for breast cancer development [19]. Currently, there is no available breast cancer gene repository assisting clinical translation; thus, we set out to build a manually curated database of breast cancer-associated genes and variants, using the flow chart outlined in Fig. 1.

The literature search yielded a multitude of publications and after manual evaluation the database was constructed based on a subset of 345 studies. Among these 345 studies, we manually extracted “rsID” and “OR” (also SIR, relative risk and cumulative risk; in relevant studies) for every reported breast cancer-associated genetic variant. Further, using the “rsID”, we manually mapped the (1) AF (GnomAD), (2) SNP location (to identify whether the mutation is located within a gene or in an intergenic region), and (3) genomic loci of every reported breast cancer-associated variant. Meanwhile, using the OR, we manually annotated every breast cancer-associated variants as either (1) Disease (OR > 1; variant that increases disease risk), (2) Protective (OR < 1; variant that confers protection against breast cancer), or (3) Both (variants that were shown to have both OR > 1 and OR < 1 in different studies). Following data extraction and annotation, we constructed the Curated Breast Cancer Gene (https://cbcg.dk) database, a freely available database for the future collation of new breast cancer-associated variants and genes.

### Demography of breast cancer-associated variants

We indexed 925 records in total; the term records instead of SNP/gene is used because similar SNPs/genes were reported multiple times to be associated with breast cancer by different studies. Same SNPs (or genes) that were reported by multiple studies were indexed separately as a unique record. Similarly, different SNPs (or genes) that were reported by a specific study were indexed separately as a unique record.

**Fig. 2:**
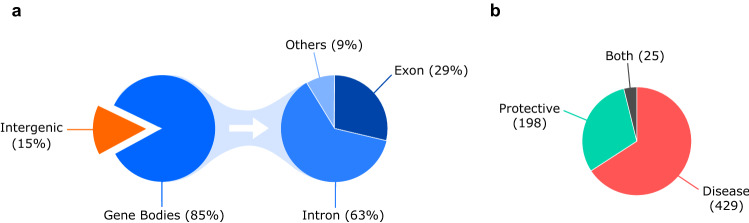
**a** Pie chart outlining the distribution of 652 breast cancer-associated loci across the genome. **b** Pie chart outlining the distribution of variants that either predisposes to breast cancer (Disease; OR > 1) or confers protection against breast cancer (Protective; OR < 1)

As depicted in Fig. 2a, we cataloged in total 652 breast cancer-associated loci across the genome. Among these, 551 breast cancer loci (85%) were located within a gene (intron, exon or UTR region, etc.). Interestingly, a large number of 101 breast cancer loci (15%) were present in the intergenic region. Of the 551 breast cancer loci located within a gene, a majority of them (345, 63%) were present in the intron. Breast cancer-associated variants were also reported in the UTR and splice site regions etc. accounting for 9% (48) of the breast cancer loci located within a gene. However, only 29% (158) of the reported breast cancer loci were located within an exonic region. Taken together, most of the reported breast cancer-associated variants (446) were present in the non-coding region such as intergenic (101) and intronic (345). SNPs located in the intergenic and intronic regions are suggested to play a role in the regulation of gene expression [20, 21].

Further, we cataloged the breast cancer-associated variants based on their OR to identify variants that either predispose to breast cancer (Disease; OR > 1) or confer protection against breast cancer (Protective; OR < 1). In our analyses, 429 breast cancer-associated variants were identified to predispose to breast cancer, whereas 198 breast cancer-associated variants were identified to confer protection against breast cancer (Fig. 2b). We also identified 25 breast cancer-associated variants that were reported to both predispose to breast cancer and confer protection against breast cancer in different studies (Fig. 2b). These conflicting results are mostly observed in studies performed in different populations, suggesting population-based effects.

### Chromosomal ideogram analysis

**Fig. 3:**
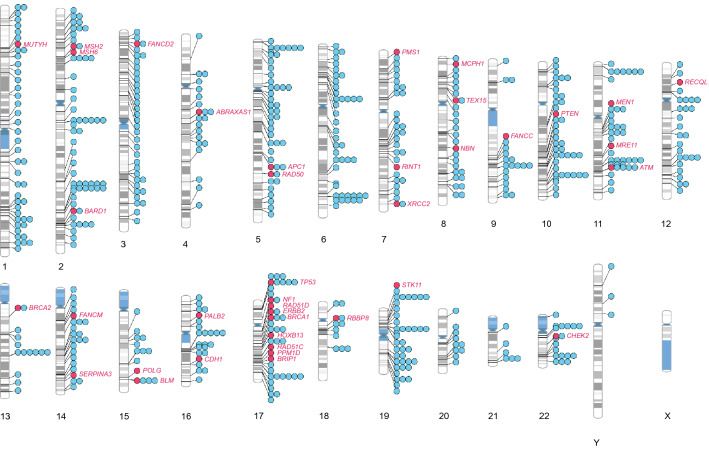
Chromosomal ideogram illustrating the distribution of 652 breast cancer-associated loci across the chromosomes. Chromosomal ideogram was constructed using PhenoGram software tool [15] with each dot representing one gene or variant

**Fig. 4:**
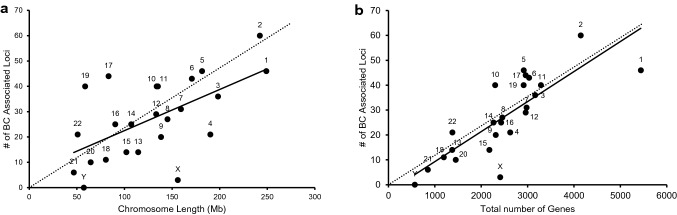
**a** Scatter plot illustrating the number of breast cancer-associated loci relative to its length for every chromosome. The chromosomal length for each chromosome was retrieved from Ensembl under Chromosome Statistics. **b** Scatter plot illustrating the number of breast cancer-associated loci relative to the total number of genes present in each chromosome. The total number of genes for each chromosome was calculated using Ensembl (Chromosome Statistics) by adding the number of coding genes, non-coding genes, and pseudogenes. The thick continuous line depicts the trendline for the number of breast cancer-associated loci present in each chromosome compared to its length (**a**) or the total number of genes present in that chromosome (**b**). **a** and **b** The thin dotted line is an imaginary trendline to illustrate a perfect positive correlation

In order to identify chromosomal regions that are enriched or devoid of breast cancer-associated variants, we performed ideogram analysis in the 652 breast cancer-associated loci (Fig. 3) [15]. The highest number of breast cancer-associated loci (60) was found on chromosome 2, whereas the lowest number of breast cancer-associated loci (6) was found on chromosome 21 (excluding sex chromosomes). Since chromosomes are of differing length, we next analyzed the number of breast cancer-associated loci relative to its length for every chromosome (Fig. 4a). Despite its larger size, the density of breast cancer-associated loci was lower in chromosome 4 (Fig. 4a). Similarly, chromosomes 17 and 19 had the highest density of breast cancer-associated loci when compared to its chromosomal size (Fig. 4a).

Since, chromosome 17 and 19 have been shown to possess the highest gene density of all human chromosomes [22, 23], we next analyzed the number of breast cancer-associated loci relative to the number of genes present in each chromosome (Fig. 4b). The presence of increased breast cancer-associated loci in chromosome 17 and 19 correlates with the presence of larger number of genes in these chromosomes (Fig. 4b).

### Manual curation of rare-monogenic variants

Breast cancer gene panels are commonly used by diagnostic laboratories to identify disease etiology among patients. The gene panels include genes with a well-documented association between a rare-monogenic variant and breast cancer (e.g., *BRCA1*, *BRCA2* and *PALB2*). Inclusion of bonafide breast cancer-associated genes in future diagnostic gene panels would increase the odds of uncovering disease etiology among patients. Hence, we manually annotated and collated the breast cancer genes in which a previous association between rare-monogenic variant and breast cancer were established.

**Fig. 5:**
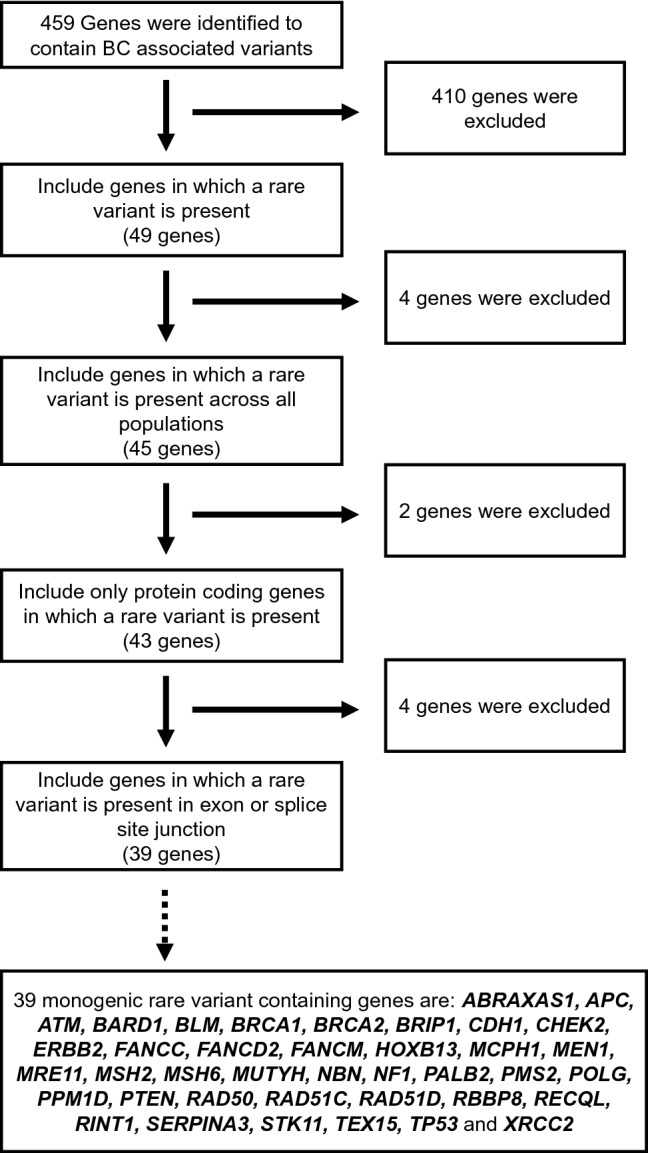
Flow chart outlining the different criteria used to annotate and collate the rare-monogenic variant containing breast cancer genes. Out of the 459 breast cancer genes, our manual curation effort has identified 39 genes to contain disease-causing monogenic variants

In total, we identified 459 genes with breast cancer-associated variants (Fig. 5). In order to annotate and collate the rare-monogenic variant containing breast cancer genes, we set out the following criteria: (1) genes should contain at least one rare variant (49 genes); (2) these rare variants should be rare across all population (45 genes); (3) these rare variants should be present in protein coding genes (43 genes); and (4) these rare variants should be present in the coding regions of a gene and not in intron (39 genes) (Fig. 5). The 39 genes that we identified to contain disease-causing monogenic variants are *ABRAXAS1, APC, ATM, BARD1, BLM, BRCA1, BRCA2, BRIP1, CDH1, CHEK2, ERBB2, FANCC, FANCD2, FANCM, HOXB13, MCPH1, MEN1, MRE11, MSH2, MSH6, MUTYH, NBN, NF1, PALB2, PMS2, POLG, PPM1D, PTEN, RAD50, RAD51C, RAD51D, RBBP8, RECQL, RINT1, SERPINA3, STK11, TEX15, TP53, and XRCC2* (Fig. 5).

A majority of these are well-known cancer syndrome genes or genes that maintain genomic stability, such breast cancer genes are marked with red dots and annotated, respectively, in Fig. 3. The majority of the 39 monogenic rare variant containing genes are either well-known tumor suppressors or suspected to have a tumor suppressor role, whereas only *PPM1D* and *ERBB2* are classified as bonafide oncogenes by the Cancer Gene Census [24]. Of note, Chromosome 17 contains many rare-monogenic variants containing breast cancer genes (Fig. 3). Using the new platform (https://cbcg.dk), this monogenic breast cancer gene list will be continually updated for clinical and diagnostic purposes.

### Gene-set enrichment analyses

**Fig. 6:**
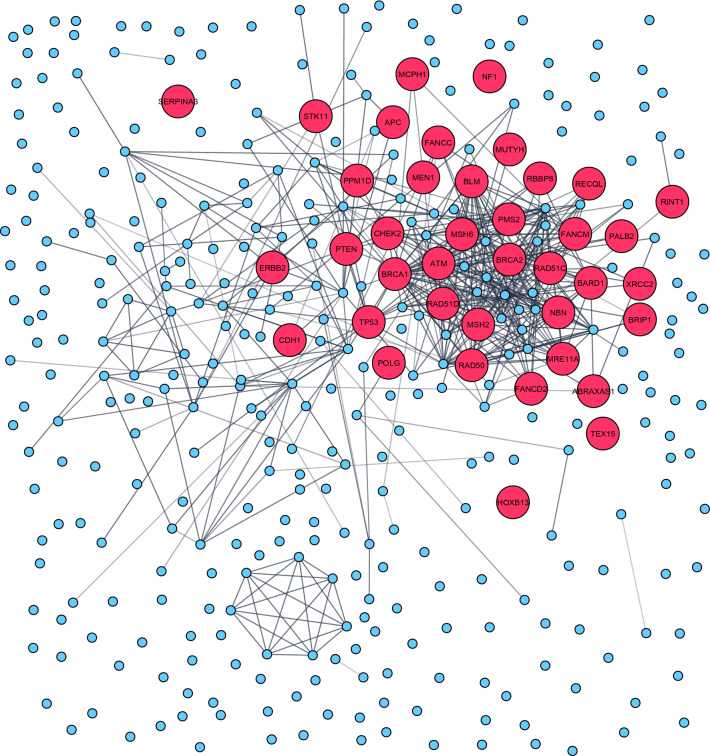
Protein network analysis performed in the 459 breast cancer genes revealed a major cluster enriched among the DNA repair pathways. Rare-monogenic variant containing breast cancer genes (red dots) was mainly present within this cluster. The protein–protein interaction network was constructed using STRING database [16] and graphically adjusted in Cytoscape [17]

To identify enrichment of specific molecular pathways and biological processes in the cataloged 459 breast cancer-associated genes, we performed both network analysis and functional enrichment analysis. The protein network analysis performed using cytoscape/String revealed a major cluster enriched among the DNA repair pathways, attributable to the rare-monogenic variant containing breast cancer genes (red dots) that were mainly present within this cluster (Fig. 6). On the contrary, a vast majority of the common-polygenic variant containing breast cancer genes (blue dots) were devoid of any protein–protein interaction and thus lacking pathway clustering (Fig. 6).

To further characterize, we next performed functional enrichment analysis using the g:Profiler [18] g:GOSt tool. In agreement with network analysis, the functional enrichment analysis (using KEGG) also indicated DNA repair pathways such as homologous recombination and Fanconi anemia to be significantly enriched among the cataloged breast cancer-associated genes (Supplementary Table 1). These DNA repair pathways together with platinum drug resistance pathway comprise the majority of annotated rare-monogenic variant containing breast cancer genes (Supplementary Table 1). However, pathways such as steroid hormone biosynthesis, chemical carcinogenesis, and metabolism of xenobiotics by cytochrome P450 were found to be significantly enriched among the common-polygenic variant containing breast cancer genes (Supplementary Table 1). Thus, the results from both network and functional enrichment analysis indicate that the rare-monogenic and common-polygenic variant containing breast cancer genes were implicated mainly in DNA repair and steroid metabolism, respectively.

## Discussion

The potential use of breast cancer genes and variants during clinical management is prodigious, thus, identification of new factors responsible for breast cancer etiology is of paramount importance. An expedited translation of these newly identified breast cancer genes and variants could greatly augment personalized breast cancer treatment. However, with a constant influx of new studies, it is challenging to rapidly integrate these new findings into clinical use. The presence of a database comprising breast cancer-associated factors would enable rapid translation into clinical diagnostics. However, to the best of our knowledge, currently there is no database that archives known breast cancer genes and variants. Thus, there is a pressing need for an interactive and accessible database of curated breast cancer susceptibility genes.

We built a database of curated breast cancer genes (https://cbcg.dk) that can be readily used by both breast cancer researchers and clinicians. The main novelty of the study and linked database are that every breast cancer genes/variant, its rsID, SNP location, genomic location, AF and whether it is a potentially disease-causing genetic variant have been carefully and stringently curated. Another novelty of this study is the compilation of 39 genes that were identified to contain disease-causing monogenic variants. We believe that this database will not only readily provide information for both breast cancer researchers and clinicians but also help in saving their time. It is our view to provide continual updates of the data repository by curating new breast cancer genes/variants, most importantly, monogenic breast cancer gene list will be continually updated for clinical and diagnostic purposes.

Identification of breast cancer-associated rare-monogenic variants are typically performed using targeted gene sequencing that utilizes a focused panel of selected genes. The genes included in these breast cancer multigene panels are different among vendors (for a list of commonly used breast cancer multigene panel, please read Easton, DF et al. [25]). The only similarity between these multigene panels is that they mainly focus on DNA repair genes, other than that, there exists no clear consensus on the design of these multigene breast cancer panels [26]. One important aspect while designing a future (or custom) breast cancer multigene panel is to consider maximizing the likelihood of uncovering breast cancer-associated rare variants among the patients. We believe that including genes in which a previous association between rare-monogenic variant and breast cancer has been documented would maximize the odds of uncovering breast cancer-associated alterations.

A list of these breast cancer genes could be also used by the clinicians to narrow their search of breast cancer-associated alteration in the WES or WGS of patient data. Currently, to the best of our knowledge there exists no curated breast cancer gene list that could facilitate the screening of rare-monogenic variants. Therefore, we manually annotated and collated 39 breast cancer genes in which a previous association between rare-monogenic variant and breast cancer has been documented. Interestingly, 28 out of these 39 breast cancer genes were included in the screening panel (comprising 34 genes) of a recent study that aimed to identify overall breast cancer risk in more than 113,000 women [27]. This further exemplifies the appropriateness of our database in clinical high-throughput sequencing approaches such as multigene panel testing or in-silico panel testing from WES or WGS platforms. As the most cost-effective sequence method is soon to be the WGS, it enables the option of increasing the *in-silico* gene panel in clinical screening of breast cancer patient. However, as recently shown from a consortium of international breast cancer genetic screening laboratories, the gene panels (in-silico or capture based) are far from compatible, nor is it possible to update with the constant flow of new knowledge [26].

Dissecting of breast cancer genes includes not only the rare-monogenic variants but also the growing number of common-polygenic variants. While the individual common-polygenic variants have small effects on disease risk, cumulatively, they can cause an increased disease risk, similar to that of rare-monogenic variant [7]. Utilizing the GWAS identified common-polygenic variants, PRS are estimated and the prospect of utilizing PRS as a clinical tool is gaining traction [28]. Already, some clinics have chosen to offer a polygenic risk calculation through commercial test laboratories [29]. Translating breast cancer-associated SNPs into clinical practice is troublesome and there is currently a considerable debate over the clinical utility of PRS to assess breast cancer risk [11]. Although evidence for support of implementation of PRS into clinical practice is sparse, there is no doubt that PRS will play an enormous role in the future population screening programs, providing healthy persons a personalized risk assessment and managing tools [30]. For researchers and stakeholders, it is possible to assess the breast cancer-associated SNPs through the GWAS Catalog (https://www.ebi.ac.uk/gwas/). We believe that the https://cbcg.dk database could also aid in the implementation of PRS into clinical practice.

There are few shortcomings in this current database mainly concerning the inability of us to provide unambiguous information about the population and breast cancer subtype for every curated breast cancer gene/variant. Moreover, during the construction of this database, we have also observed a great disparity between study populations among the enrolled studies, with most involving European/Caucasian patients. The genetic discovery efforts to date heavily underrepresent non-European populations globally and this has serious impact during PRS estimation in non-European patients. It has been shown several times that PRS predicts individual risk far more accurately in Europeans when compared to non-Europeans due to the overwhelming abundance of GWAS studies conducted in participants of European descent [31].

It is by now well established that the majority of known rare causal germline breast cancer genes are involved in genome maintenances pathways (Fig. 6). However, when searching for new causal breast cancer genes it is relevant to unravel if entirely new or interacting pathways are potential areas to seek for causal monogenic variants. We believe that our database could serve as an inspiration to find these new pathways where new breast cancer causal genes could function. Keeping this in mind, we have built an interactive and accessible database of curated breast cancer genes (https://cbcg.dk), to support the ongoing efforts in managing breast cancer etiology.

## Supplementary Information

Below is the link to the electronic supplementary material.

Supplementary Table 1: Functional enrichment analysis performed in the 459 breast cancer genes using the g:Profiler [18] g:GOSt tool. Results from the analysis performed in data sources such as GO MF, GO CC, GO BP, KEGG, Reactome and WikiPathways are reported in separate tabs. (XLSX 348 kb)

## Data Availability

The datasets generated during and/or analyzed during the current study are available in the [https://cbcg.dk] repository.
